# 723 Effectiveness and Efficiency of Massage Therapy for the Alleviation of Pain in Pediatric Burn Patients

**DOI:** 10.1093/jbcr/irae036.266

**Published:** 2024-04-17

**Authors:** Ben T Reader, Deborah Zerkle, Rajan K Thakkar, Dana M Schwartz

**Affiliations:** Nationwide Children's Hospital, Columbus, OH; Nationwide Children's Hospital, Columbus, OH; Nationwide Children's Hospital, Columbus, OH; Nationwide Children's Hospital, Columbus, OH

## Abstract

**Introduction:**

Comprehensive patient-centered burn care requires not only treating burn wounds, but also addressing the stress, pain, and anxiety that burn injuries incite. Massage therapy (MT) has demonstrated promising results in the adult literature in reducing pain, alleviating anxiety, and enhancing patient satisfaction. Utilization of MT in pediatric acute burn patients is a novel approach; we sought to examine the utilization of MT and its impact on pain in this population.

**Methods:**

A retrospective review of electronic health records was conducted for pediatric burn patients aged 0-18 years admitted to the inpatient floor our ABA-verified Pediatric Burn Center between January 2022 to July 2023. The primary objective was to evaluate the efficiency and effectiveness of administration of MT as part of inpatient burn admissions. Patients admitted to the intensive care unit were excluded. Descriptive statistics summarized patient demographics, injury details, and variables related to MT treatment. To investigate the potential impact of age, length of stay (LOS), and total body surface area (TBSA) on the provision of MT, a binary logistic regression was performed, and a Bonferroni correction was applied to control for multiple comparisons.

**Results:**

A total of 198 patients meeting the inclusion criteria were identified, with 100% rate of MT consultation on admission. Twenty-seven (13.6%) unique patients received a combined total of 43 MT sessions. Median session duration was 24.5 minutes (IQR: 7 minutes) and typical MT frequency was 2-3 sessions per week. Burn mechanisms in the MT group included scalds (55.6%), flame (22.2%), and contact (14.8%) burns.

Of treated patients, 12 patients reported pain prior to MT delivery (28.6%) and 3 patient reported persistent pain after MT delivery, representing a 75% reduction in patient-reported pain. After MT, 95.3% of patients were observed to be content/relaxed or resting comfortably. Of patients not treated by MT, 20.5% were attempted at least once. Barriers to MT delivery included patients asleep (42.1%), patients off unit (33.7%), and patients attended to by other healthcare providers (21.1%). Of patients with no recorded MT treatment attempt (n=136), 49.6% of admissions occurred on Friday, Saturday, or Sunday. Compared to patients who received MT, patients who did not have MT had shorter median LOS (1 day [IQR:1 day] vs 4 days [IQR: 5 days] (p=.036).

**Conclusions:**

Massage Therapy for children admitted with acute burns reduced pain and promoted contentment/relaxation. Patients admitted on weekends and with short LOS frequently missed MT treatment. Additional weekend resources, education of providers on MT benefits, and increasing frequency of patient checks for MT-readiness would increase access to this beneficial resource.

**Applicability of Research to Practice:**

Implementing MT for pediatric burn patients is not only feasible but also effective in reducing pain and enhancing relaxation, making it a valuable addition to practice.

